# Comorbid vision and cognitive impairments in older adults hospitalized for acute myocardial infarction

**DOI:** 10.1177/2235042X20940493

**Published:** 2020-07-16

**Authors:** Heather E Whitson, Alexandra M Hajduk, Xuemei Song, Mary Geda, Sui Tsang, John Brush, Sarwat I Chaudhry

**Affiliations:** 1 Department of Medicine, Duke University School of Medicine, Durham, NC, USA; 2 Department of Ophthalmology, Duke University School of Medicine, Durham, NC, USA; 3 Center for the Study of Aging and Human Development, Duke University School of Medicine, Durham, NC, USA; 4 Geriatrics Research Education and Clinical Center, Durham Veterans Administration Medical Center, Durham, NC, USA; 5 Department of Medicine, Yale University School of Medicine, New Haven, CT, USA; 6 Yale University Program on Aging, New Haven, CT, USA; 7 Sentara Healthcare, Norfolk, VA, USA

**Keywords:** Vision, cognition, cardiovascular disease, hospital, aging

## Abstract

Older patients presenting with acute myocardial infarction (AMI) often have comorbidities. Our objective was to examine how outcomes differ by cognitive and vision status in older AMI patients. We use data from a prospective cohort study conducted at 94 hospitals in the United States between January 2013 and October 2016 that enrolled men and women aged ≥75 years with AMI. Cognitive impairment (CI) was defined as telephone interview for cognitive status (TICS) score <27; vision impairment (VI) and activities of daily living (ADLs) were assessed by questionnaire. Of 2988 senior AMI patients, 260 (8.7%) had CI but no VI, 858 (28.7%) had VI but no CI, and 251 (8.4%) had both CI/VI. Patients in the VI/CI group were most likely to exhibit geriatric syndromes. More severe VI was associated with lower (worse) scores on the TICS (*β* −1.53, 95% confidence interval (CI) −1.87 to −1.18). In adjusted models, compared to participants with neither impairment, participants with VI/CI were more likely to die (hazard ratio 1.61, 95% CI 1.10–2.37) and experience ADL decline (odds ratio 2.11, 95% CI 1.39–3.21) at 180 days. Comorbid CIs and VIs were associated with high rates of death and worsening disability after discharge among seniors hospitalized for AMI. Future research should evaluate protocols to accommodate these impairments during AMI presentations and optimize decision-making and outcomes.

## Introduction

The most dominant risk factor for coronary heart disease (CHD) is age.^1,2^ The prevalence of CHD among those aged 60–79 years for men and women, respectively, is 15.1% and 24.4%, and among those aged 80 years and over, CHD prevalence is 23.9% and 36.1%.^3^ Additionally, life expectancy has increased, and the average age of the CHD patient population is rising.^4^ The complexity of an aging patient population poses new challenges in cardiovascular care.^5,6^ Patients with heart disease frequently experience age-related syndromes, including comorbidity, frailty, malnutrition, and cognitive impairment (CI) and sensory impairment.^7–10^ Easily administered tools that assess geriatric health factors, such as nutrition and physical performance, improve the ability of traditional risk stratification systems (e.g. the Global Registry of Acute Coronary Events risk score) to predict 1-year mortality in older adults with acute coronary events.^10,11^ In acute CHD presentations, such as acute myocardial infarction (AMI), when potentially life-altering treatment decisions must be made quickly, it can be difficult to navigate care options for patients with certain age-related conditions.^12–14^

Two age-related conditions that may impact care decisions and health outcomes are impairments in cognition and vision.^15–18^ Previous studies in clinical populations, such as chronic obstructive pulmonary disease and heart failure patients, demonstrate that treatment outcomes differ according to comorbid conditions.^19–21^ One reason to focus on CI and vision impairment (VI) in the AMI population is that these impairments share risk factors related to vascular disease,^22–25^ so are likely to be common among seniors hospitalized with AMI. Another reason is that care and outcomes related to AMI hospitalization could be influenced by vision and cognitive status. These impairments may limit patient–provider communication, which is critical when care teams are making time-sensitive decisions that are aligned with the preferences of complex patients. Additionally, VI and CI could interfere with patients’ ability to manage their health needs after AMI hospitalization, which could adversely affect outcomes.

In this analysis, we use data from the Comprehensive Evaluation of Risk Factors in Older Patients with acute myocardial infarction (SILVER-AMI) study, an observational, multicenter study of older Americans (aged ≥75 years) hospitalized with AMI. There are over 700,000 AMI hospitalizations annually in the United States, and the majority of AMIs affect adults over age 65.^26,27^ The primary objective of the current analysis was to compare care and outcomes after AMI hospitalization in seniors with and without comorbid VI and CI. This objective was motivated by our desire to identify opportunities to improve AMI prognosis and management for individuals with these impairments.

A secondary objective was to examine the relationship between CI and VI in this population. The relationship between CI and VI in people with AMI is of interest because prior research in community cohorts suggests that CI and VI co-occur more frequently than would be expected by chance.^25,28–30^ Several mechanisms may contribute to this association, and one possibility is that vascular risk factors predispose individuals to organ damage that arises concurrently in brain and eyes.^18^ It is therefore of interest to determine whether a relationship exists between VI and CI within a population that is defined by cardiovascular disease. The SILVER-AMI study provides an opportunity to further our understanding of the epidemiological relationship between vision and cognition and to define care patterns in older patients with both impairments.

## Methods

### Study population

SILVER-AMI is a longitudinal cohort study, which enrolled 3041 participants aged ≥75 years, who presented to one of 94 hospitals in the United States between January 2013 and October 2016 with an AMI. Verified outcomes of interest for the current analysis were available in 2988 (98.4%) SILVER-AMI participants, who represent our study cohort. Design and rationale of SILVER-AMI have been reported in detail elsewhere.^31^ Briefly, research coordinators at each site reviewed daily admission records to identify and screen potentially eligible participants. Inclusion criteria were aged ≥75 years and diagnosed with AMI in accordance with the universal definition of myocardial infarction (MI).^32^ Participants were excluded whether they were transferred from another hospital, incarcerated, unable to provide consent and lacked available proxy, experienced an MI attributed to inpatient surgery or procedure, or whether the initial troponin elevation occurred >24 h after admission. All participants provided informed consent and SILVER-AMI was approved by Institutional Review Boards at all recruitment and analysis sites. The data that support the findings of this study are available from the executive body of the SILVER-AMI study at Yale University School of Medicine. Restrictions apply to the availability of data, as primary analyses are ongoing by study investigators. Data and SAS code are available upon request with the permission of the executive body of SILVER-AMI. Requests should be made to principal investigator SIC (sarwat.chaudhry@yale.edu).

### Data collection

SILVER-AMI participants participated in a baseline interview and assessment during AMI hospitalization and a telephone interview 6 months after discharge. Additional clinical data were collected by trained study staff from medical record abstraction, including records from the baseline AMI hospitalization as well as any subsequent hospitalizations or emergency department visits during the following 6 months.

### Cognitive impairment

Cognitive status was assessed with the telephone interview for cognitive status (TICS)^33^ at the baseline interview. The TICS is a validated global test of cognition, which can be administered in <10 min, is used to detect both mild and severe CIs, and does not require reading or writing (i.e. is not dependent on visual or motor abilities). CI was defined as present if the participant had a TICS score of <27, which has been reported as an ideal cut point for distinguishing individuals with normal versus impaired cognition^34^ and corresponds approximately to the widely used mini mental state examination cut point of <24.^35^

### Vision impairment

Visual ability was assessed with a question from the National Eye Institute Vision Functional Questionnaire^36^: “At the present time, would you say your eyesight using both eyes is excellent, good, fair, poor, very poor, or are you completely blind?” Visual impairment was defined as a response of fair, poor, very poor, or completely blind. Self-report of vision status has been shown to correlate well to measures of visual acuity, contrast sensitivity, stereo acuity, and (to a lesser degree) visual fields.^37^

### Main outcomes

At the 6-month review, deaths were confirmed by death certificate, medical record review, or in rare cases, obituary. Readmissions to any hospital within 180 days from the index discharge were adjudicated after reconciling patient/proxy reports in the 6-month interview with medical record review. Preadmission activities of daily living (ADLs) were assessed with four questions about the ability to perform, without help from another person, bathing, dressing, getting out of a chair, and ambulating.^38^ The ADL survey was repeated at the 6-month interview and compared to baseline to determine whether there had been a decline in functional status.

### Other measures

Age was calculated from birth year, as reported in medical records. Each participant’s race, sex, marital status, highest attained education level, living arrangement, presence or absence of unintentional weight loss of more than 10 pounds in the previous year, and smoking status were assessed in the baseline interview. Depression symptoms were assessed with the eight-item Patient Health Questionnaire^39^ and score >10 was defined as a positive screen for depression.

Clinical variables at the time of the initial presentation were abstracted from health records: blood pressure, heart rate, Killip class,^40^ time from symptom onset to presentation, MI classification (ST elevation MI (STEMI) or not), left ventricular ejection fraction, comorbidities, laboratory results, in-hospital complications, and discharge disposition. The Charlson comorbidity index score was calculated based on information about comorbidities.^41^ Information about revascularization during the hospitalization, cardiac procedures, and discharge instructions were abstracted from health records.

### Statistical analysis

#### Examining relationship between cognition and vision

We constructed linear regression models to estimate the relationship between the ordinal vision variable (six levels, excellent to completely blind, with higher values indicating worse vision) as the primary independent variable and TICS score (higher values indicating better cognition). We evaluated whether the relationship was attenuated after adjustment for demographics (age, sex, race, education, and living status) (model 1) or model 1 variables + vascular risk factors (diabetes mellitus, hypertension (HTN), dyslipidemia, body mass index (BMI), smoking status) (model 2) or model 2 variables + additional health variables (congestive heart failure, COPD, asthma, chronic kidney disease) (model 3). We constructed a line plot to visualize the relationship between the VI variable and TICS score.

#### Comparing care and outcomes according to vision and cognitive status

Participants were grouped into four mutually exclusive categories based on self-reported vision and TICS score: vision impairment and cognitive impairment (VI/CI), vision impairment and no cognitive impairment (VI/noCI), cognitive impairment and no vision impairment (noVI/CI), no vision impairment and no cognitive impairment (noVI/noCI). Descriptive statistics were used to characterize the overall cohort and each VI/CI category with respect to baseline variables, method of revascularization, and outcomes from the index hospitalization. Analysis of variance was used to compare means of continuous variables, and McNemar’s test was used to compare proportions of dichotomous and categorical variables across the four categories of VI/CI status.

To estimate the relationship between VI/CI status and 6-month outcomes, we constructed models with the VI/CI variable as the main predictor variable and each of the following as dependent variables: death at 180 days, readmissions at 180 days, and ADL decline (from baseline to 6 months). We used proportional hazard models to examine the relationship between VI/CI status and time to 180-day outcomes, and we used logistic regression to model the association between VI/CI status and ADL decline. Using the noVI/noCI participants as the reference group, we calculated the odds or hazards of each outcome for those with VI/no CI, with CI/no VI, or comorbid VI/CI. For each relationship, we also constructed an adjusted model with covariates known or suspected to be associated with post-AMI outcomes,^42,43^ including age, sex, race, education, living status, Charlson comorbidity index, Killip class, presenting heart rate, presenting systolic pressure, time to presentation, AMI type (STEMI/NSTEMI), length of admission, initial hemoglobin, in-hospital revascularization (none, catheterization only, percutaneous coronary intervention (PCI), coronary artery bypass graft (CABG)), in-hospital complications, ejection fraction, and discharge location. The model that predicted ADL decline also adjusted for baseline ADL status, but that model did not adjust for “discharge location” because we theorized that participation in inpatient rehabilitation after discharge could play a causative role in 6-month ADL outcome.

## Results

**Figure 1.**
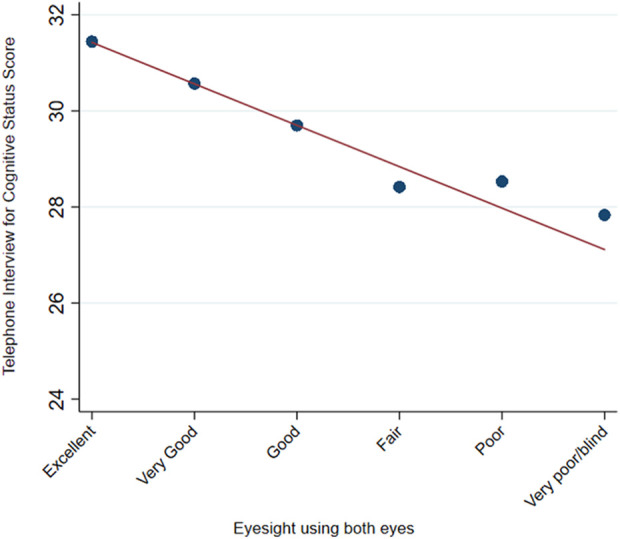
Relationship between self-reported vision status and cognitive score line graph demonstrating the relationship between participants’ self-reported vision status and average scores on the telephone interview for cognitive status screening instrument.

Table 1 presented data on the association between vision and cognitive performance, before and after adjustment for vascular risk factors. More severe VI was associated with lower (worse) scores on the TICS (*β* −1.53, 95% confidence interval (CI) −1.87 to −1.18). The strength of the association was reduced by adjustment for demographic variables (model 1) and further reduced by adjusting for cardiovascular risk and conditions (models 2 and 3). However, even in the fully adjusted models, a significant relationship existed between severity of VI and CI, as measured by the TICS (*β* −0.97, 95% CI −1.29 to −0.65). The relationship between vision status and TICS score is shown in Figure 1.

**Table 1.** Association between vision and cognitive variables among seniors presenting for AMI.

	TICS score (dependent variable)^a^			
	Unadjusted model *β* (95% CI)	Model 1 *β* (95% CI)	Model 2 *β* (95% CI)	Model 3 *β* (95% CI)
Vision impairment severity3	−1.53 (−1.87 to −1.18)	−1.03 (−1.35 to −0.71)	−0.99 (−1.31 to −0.67)	−0.97 (−1.29 to −0.65)

AMI: acute myocardial infarction; TICS: telephone interview for cognitive status; CI: confidence interval.

^a^ Higher cognitive test scores indicate better cognitive function, whereas higher vision impairment scores indicate worsening eyesight on a scale of 1 (excellent) to 6 (completely blind). Thus, negative correlations indicate that vision impairment is associated with cognitive impairment. Model 1 adjusted for age, sex, race, education (<12 years), living alone. Model 2 adjusted for model 1 variables + the following vascular disease risk factors: body mass index, smoking (current or past smoker), history of hypertension, history of diabetes, history of dyslipidemia. Model 3 adjusted for model 2 variables + the following vascular conditions: prior coronary artery disease, prior MI, prior revascularization procedure, history of peripheral artery disease, and history of cerebrovascular disease.

Of 2988 participants in the analysis, CI was detected in 511 (17.1%) and VI was reported by 1109 (37.1%). The cohort was stratified into four groups based on VI/CI status: 1619 (54.2%) participants had neither VI nor CI, 260 (8.7%) had CI but no VI, 858 (28.7%) had VI but no CI, and 251 (8.4%) had comorbid impairments in vision and cognition. In those with VI, the prevalence of CI was 22.6% (251 or 1109); in those with CI, the prevalence of VI was 49.1% (251/511).

Table 2 summarizes the characteristics of the study participants at their index hospitalization for AMI, stratified by VI/CI status. Participants in the CI groups, compared to the cognitively normal groups (regardless of vision status), tended to be older, more often female, more likely to have less than 12 years of education, and to have history of hypertension and slightly lower BMIs. By definition, those with CI also had lower scores on the TICS. Compared to all other groups, individuals with comorbid VI/CI were more often non-White and had significantly higher Charlson comorbidity scores. Individuals in the VI/CI group, compared to other groups, also had the highest rates of the following risk conditions: diabetes, dyslipidemia, history of cerebrovascular disease, and prior MI/revascularization. Both of the cognitively impaired groups, compared to the cognitively intact groups, exhibited higher risk Killip class and lower hemoglobin values at presentation. Although the mean age of all participants was over 80 years and the mean age was similar for the two groups with CI (83 years), the highest occurrence of the following age-associated risk factors, or geriatric syndromes, was seen in the group with comorbid VI/CI: positive depression screen (26.7%), unintentional weight loss of 10 pounds or more in the last year (36.3%), and ADL impairment (37.6%).

**Table 2.** Characteristics of participants presenting with AMI according to cognitive and vision status.

Characteristic	VI/CI (*N* = 251)	VI/noCI (*N* = 858)	noVI/CI (*N* = 260)	NoVI/noCI (*N* = 1619)	Total (*N* = 2988)	*p* Value
Age, mean (SD)	83.61 (5.92)	81.60 (4.98)	83.09 (5.40)	81.03 (4.72)	81.59 (5.03)	<0.001
Sex, *N* (%) female	0135 (53.78%)	0387 (45.10%)	0141 (54.23%)	0648 (40.02%)	1311 (43.88%)	<0.001
Race, *N* (%) non-White	0065 (26.42%)	0092 (10.79%)	0049 (19.37%)	0110 (06.93%)	0316 (10.58%)	<0.001
Education, *N* (%) ≤12 years	0186 (74.70%)	0486 (57.31%)	0192 (75.89%)	0826 (51.18%)	1690 (56.56%)	<0.001
Live alone	0100 (39.84%)	0342 (39.86%)	0099 (38.08%)	0602 (37.23%)	1143 (38.25%)	0.59
TICS total score						
Mean (SD)	22.44 (3.90)	31.53 (2.75)	22.59 (3.58)	32.29 (2.75)	30.40 (4.65)	<0.001
Median (range)	24.0 (6.0 – 26.0)	32.0 (27.0 – 38.0)	24.0 (6.0 – 26.0)	32.0 (27.0 – 41.0)	31.0 (6.0 – 41.0)	<0.001
Charlson comorbidity score, mean (SD)	4.30 (2.43)	3.80 (2.75)	3.90 (2.74)	3.23 (2.48)	3.54 (2.60)	<0.001
Body mass index, mean (SD)	26.93 (6.29)	27.37 (5.43)	26.72 (5.31)	27.70 (5.01)	27.46 (5.28)	0.010
Current or ever smoker, *N* (%)	0132 (53.88%)	0499 (58.43%)	0127 (49.61%)	0901 (55.96%)	1659 (55.52%)	0.08
Vascular risk conditions						
DM, *N* (%)	0124 (49.40%)	0329 (38.34%)	0114 (43.85%)	0541 (33.42%)	1108 (37.08%)	<0.001
Hypertension, *N* (%)	0226 (90.04%)	0720 (83.92%)	0236 (90.77%)	1362 (84.13%)	2544 (85.14%)	0.003
Dyslipidemia, *N* (%)	0174 (69.32%)	0525 (61.19%)	0165 (63.46%)	1013 (62.57%)	1877 (62.82%)	0.13
Cerebrovascular disease, *N* (%)	0067 (26.69%)	0128 (14.92%)	0059 (22.69%)	0212 (13.09%)	0466 (15.60%)	<0.001
Peripheral artery disease, *N* (%)	0036 (14.34%)	0112 (13.05%)	0035 (13.46%)	0174 (10.75%)	0357 (11.95%)	0.16
Prior history of coronary artery disease, *N* (%)	0150 (59.76%)	0472 (55.01%)	0138 (53.08%)	0832 (51.39%)	1592 (53.28%)	0.06
Prior MI, *N* (%)	0082 (32.67%)	0253 (29.49%)	0066 (25.38%)	0410 (25.32%)	0811 (27.14%)	0.025
Prior revascularization, *N* (%)	0121 (48.21%)	0365 (42.54%)	0100 (38.46%)	0628 (38.79%)	1214 (40.63%)	0.018
Other risk factors at presentation of AMI						
Killip class II, III, or IV, *N* (%)	0040 (15.94%)	0113 (13.17%)	0049 (18.85%)	0184 (11.37%)	0386 (12.92%)	0.003
Presenting heart rate, mean (SD)	85.8 (20.56)	84.4 (23.38)	86.2 (21.02)	82.4 (22.71)	83.6 (22.62)	0.011
Presenting systolic BP, mean (SD)	148.6 (32.37)	145.7 (30.13)	141.6 (30.25)	146.0 (30.97)	145.7 (30.81)	0.073
MI classification, STEMI, *N* (%)	0060 (23.90%)	0225 (26.22%)	0070 (26.92%)	0426 (26.31%)	0781 (26.14%)	0.86
Initial hemoglobin value, mean (SD)	12.31 (2.05)	12.74 (2.10)	12.39 (1.98)	13.03 (2.04)	12.83 (2.06)	<0.001
Left ventricular ejection fraction category, *N* (%)						0.127
≥50%	115 (45.8%)	425 (49.5%)	120 46.2%)	845 (52.2%)	1505 (50.4%)	
40–50%	56 (22.3%)	174 (20.3%)	49 (18.8%)	314 (19.4%)	593 (19.8%)	
30–40%	33 (13.1%)	118 (13.8%)	44 (16.9%)	200 (12.4%)	395 (13.2%)	
<30%	28 (11.2%)	60 (7.0%)	21 (8.1%)	110 (6.8%)	219 (7.3%)	
Time from symptom onset to presentation, *N* (%)						0.057
<6 h	132 (52.6%)	471 (54.9%)	143 (55.0%)	964 (59.5%)	1710 (57.2%)	
≥6 h to <12 h	34 (13.5%)	113 (13.2%)	25 (9.6%)	164 (10.1%)	336 (11.2%)	
≥12 h	85 (33.9%)	266 (31.0%)	90 (34.6%)	485 (30.0%)	926 (31.0%)	
Geriatric syndromes						
Depression screen positive, *N* (%)	0062 (26.96%)	0187 (22.56%)	0036 (14.75%)	0134 (08.42%)	0419 (14.02%)	<0.001
Unintentional weight loss, *N* (%)	0090 (36.29%)	0223 (26.05%)	0083 (32.42%)	0274 (16.99%)	0670 (22.42%)	<0.001
Preadmission ADL impairment	0094 (37.60%)	0137 (15.97%)	0054 (20.77%)	0120 (07.41%)	0405 (13.55%)	<0.001

AMI: acute myocardial infarction; TICS: telephone interview for cognitive status; DM: diabetes mellitus; ADL: activity of daily living; STEMI: ST elevation myocardial infarction; MI: myocardial infarction; VI: vision impairment; CI: cognitive impairment; SD: standard deviation.

Table 3 summarizes revascularization patterns and care and outcomes related to the index hospitalization for AMI among participants in each VI/CI category. There were significant differences across the groups in regard to receipt of procedures for revascularization. In the cognitively impaired groups, only 59.8% (VI/CI) and 59.2% (noVI/CI) of participants underwent either PCI or CABG prior to discharge, as compared to 67.1% (VI/noCI) and 73.5% (no VI/no CI) in the cognitively intact groups. Individuals with comorbid VI/CI were the most likely to receive only medical management (29.1%, as compared to 15.1% in the full cohort and 11.7% among patients with neither VI nor CI) and the least likely to undergo CABG (9.2%, compared to 11.9% in the full cohort and 12.6% of participants with neither VI nor CI).

**Table 3.** Treatment patterns and hospital outcomes, stratified by vision and cognition status, among seniors presenting with AMI.

	VI/CI (*N* = 251)	VI/noCI (*N* = 858)	noVI/CI (*N* = 260)	noVI/noCI (*N* = 1619)	Total (*N* = 2988)	*p* Value
Revascularization						
Medical management only	0073 (29.1%)	0128 (14.9%)	0061 (23.5%)	0189 (11.7%)	0451 (15.1%)	<0.001
Coronary angiography but no revascularization	0030 (12.0%)	0165 (19.2%)	0048 (18.5%)	0255 (15.8%)	0498 (16.7%)	0.02
PCI	0127 (50.6%)	0480 (55.9%)	0125 (48.1%)	0981 (60.6%)	1713 (57.3%)	<0.001
CABG performed	0023 (9.2%)	0096 (11.2%)	0029 (11.2%)	0208 (12.9%)	0356 (11.9%)	0.29
Hospital outcomes						
Any complication^a^	0170 (67.7%)	0518 (60.5%)	0168 (64.6%)	0913 (56.4%)	1769 (59.2%)	<0.001
Died in hospital	04 (1.6%)	08 (0.9%)	002 (0.8%)	0020 (1.2%)	0034 (1.1%)	0.76
Length of stay, mean (SD)	7.0 (6.8)	5.8 (4.9)	7.6 (6.8)	5.7 (5.3)	6.0 (5.5)	<0.001
Cardiac rehab program after discharge	0044 (27.7%)	0251 (37.4%)	0034 (19.7%)	0595 (44.2%)	0924 (30.9%)	<0.001
Discharge to home	0159 (64.4%)	0689 (81.1%)	0171 (66.3%)	1361 (85.1%)	2380 (79.7%)	<0.001

SD: standard deviation; PCI: percutaneous intervention; CABG: coronary artery bypass graft; AMI: acute myocardial infarction; VI: vision impairment; CI: cognitive impairment.

^a^In-hospital complications abstracted from the medical record include heart failure, cardiogenic shock, bleeding, stroke, AKI, blood transfusion, and so on.

Only 34 (1.1%) of the participants died during their hospitalization and rates of in-hospital death did not differ significantly across VI/CI groups. There were significant differences across groups in rates of complications, length of stay, rates of discharge to home, and receipt of cardiac rehabilitation after discharge (*p* < 0.001 for all), with those in the cognitively impaired groups more likely to experience complications and longer hospitalizations and less likely to be discharged home or to participate in cardiac rehab.

Our main objective was to evaluate whether VI/CI status was predictive of 180-day outcomes in this cohort. In the 180 days after discharge, there were 258 deaths (8.6%) and 1203 (40.3%) participants were readmitted. Results of proportional hazard models are summarized in Table 4. Compared to participants with neither CI nor VI, participants with comorbid VI and CI experienced higher hazard of death (hazard ratio (HR) 2.81, 95% CI 2.02–3.91) and readmission (HR 1.28, 95% CI 1.05–1.57). The hazard of death in this group remained significantly elevated, even after adjustment for multiple potential confounders (HR 1.61, 95% CI 1.10–2.37). Compared to participants without VI or CI, participants with CI (without VI) exhibited higher hazard of death (HR 2.54, 95% CI 1.81–3.57) and readmission (HR 1.58, 95% CI 1.31 -1.90). Even in the fully adjusted model, this group remained at significantly higher hazard of death (HR 1.56, 95% CI 1.07–2.27) and readmission (HR 1.21, 95% CI 1.00–1.48). Participants with VI (but not CI) had 15% increased hazard of hospital readmission (HR 1.15, 95% CI 1.01–1.31), but this was attenuated in the adjusted models.

**Table 4.** Association of vision/cognition status with 180-day outcomes, following presentation for AMI.^a^

Outcome	Unadjusted model HR (95% CI)	Adjusted model HR (95% CI)
Death		
VI/CI	2.81 (2.02–3.91)	1.61 (1.10–2.37)
VI/noCI	1.31 (0.99–1.73)	1.13 (0.83–1.53)
noVI/CI	2.54 (1.81–3.57)	1.56 (1.07–2.27)
Readmissions		
VI/CI	1.28 (1.05–1.57)	1.00 (0.81–1.23)
VI/noCI	1.15 (1.01–1.31)	1.03 (0.91–1.18)
noVI/CI	1.58 (1.31–1.90)	1.21 (1.00–1.48)
	Unadjusted model OR (95% CI)	Adjusted model OR (95% CI)
ADL decline		
VI/CI	3.63 (2.52–5.22)	2.11 (1.39–3.21)
VI/noCI	1.29 (0.98–1.70)	1.02 (0.76–1.37)
noVI/CI	1.97 (1.32–2.94)	1.15 (0.74–1.80)

AMI: acute myocardial infarction; VI: vision impairment; CI: cognitive impairment; STEMI: ST elevation myocardial infarction; NSTEMI: non-ST elevation myocardial infarction; ADL: activity of daily living; HR: hazard ratio; OR: odds ratio.

^a^ Reference group = no VI/no CI. Adjusted model includes age, sex, race, education (<12 years), living alone, Charlson comorbidity score, Killip class, initial heart rate, initial systolic blood pressure, time from symptom onset to presentation, STEMI versus NSTEMI, length of stay, initial hemoglobin value, LV ejection fraction, revascularization received, in-hospital complications, and discharge location. In the model in which the dependent variable is “ADL decline,” the adjusted model excludes “discharge location,” as we theorized that participation in inpatient rehabilitation after hospitalization could play a causative role in future ADL performance. This model instead includes a variable for baseline ADL status.

Table 4 also summarizes results of regression models to estimate the odds of worsening disability. Compared to the group with neither VI nor CI, there were higher odds of ADL decline among those with CI and no VI (odds ratio (OR) 1.97, 95% CI 1.32–2.94), and the odds of ADL decline were over three times as high among those with comorbid VI/CI (OR 3.63, 95% CI 2.52–5.22). The odds of ADL decline in the CI/noVI group were attenuated in the adjusted model, but the odds of ADL decline in the VI/CI group remained significantly elevated (OR 2.11, 95% CI 1.39–3.21).

## Discussion

Among older patients presenting to American hospitals with AMI, over 45% had easily detectable impairments in vision or cognition or both. The presence of these impairments identified patient groups at particularly high risk of adverse outcomes during the index hospitalization and over the next 6 months. Patients with VI and CI had many risk factors for adverse outcomes with sociodemographic disparities, particularly evident among those with CI, regardless of vision status. Individuals with comorbid VI and CI presented with high rates of geriatric syndromes, such as comorbidity, weight loss (a feature of the frailty syndrome),^44^ and disability. Although all participants with CI tended to have lower BMIs than the cognitively intact participants, the group with comorbid VI and CI was especially likely to report unintentional weight loss. Unintentional weight loss can be a sign of poor nutrition which, along with other frailty syndrome features, has been associated with high risk in cardiac patients.^10,45^ Even after rigorous adjustment for potential confounders, AMI patients with comorbid VI and CI, compared to those with intact cognition and vision, were at significantly higher risk of death and functional decline.

Our results provide useful new information about the frequency of concurrent VI and CI among older AMI patients. In this national population of hospitalized AMI patients over age 75 years, about 1 in 12 had both VI and CI. Both CI and VI are “invisible” problems that are often underdiagnosed in clinical settings.^16,46^ Although accommodations can be made to minimize the impact of CI and VI on patient experience, unaccommodated impairments have a negative impact on patients’ ability to communicate with providers and comply with recommendations.^47–49^ Acute settings must be prepared to screen for and accommodate these impairments in geriatric patients, regardless of the presenting complaint.

As third party payers move toward value-based care and bundled payments for health events, such as AMI, it is important to understand how comorbidities may influence care needs and utilization patterns in the geriatric population. Previous research in the community has shown that people with concurrent VI and CI are more likely to have disability, compared to peers with either single impairment.^50^ Similarly, we found that AMI patients with both VI and CI, compared to those with neither impairment, were more than three times as likely to have functionally declined 6 months after their heart attack. Additionally, we found that CI was associated with higher utilization in the 6 months after the AMI, regardless of vision status and after adjusting for many potential confounders.

Our results align with previous findings that older patients with VI exhibit lower performance on cognitive tests.^18,25,28,30^ The SILVER-AMI study offered a novel opportunity to explore the relationship between vision and cognitive status in a population of older adults defined by vascular disease, a common risk factor for both impairments.^18^ Our finding that the severity of CI was related to worsening vision in this cohort, even after adjustment for demographics and vascular risk factors and conditions suggests that additional mechanisms contribute to the observed relationship between vision health and cognition. A further strength of this analysis is that the TICS, which is the cognitive assessment used in the SILVER-AMI study, does not require visual abilities or visual cueing (e.g. no drawing, reading, or recognizing symbols), such that worse cognitive performance among visually impaired patients in this study is not attributable to testing artifact. Another advantage of the TICS is that it is relatively brief (about 10 min to administer), which is an important consideration for providers seeking tools to measure informative prognostic indicators in geriatric patients in emergency settings, such as AMI.^51^

Our study has limitations that affect the interpretation of results. First, vision was assessed by self-report. While self-reported vision has been shown to correlate well to measured visual abilities^37^ and people with CI accurately report some symptoms,^52^ our use of self-reported vision may introduce a bias. Second, to examine baseline health status and risk factors at presentation for AMI, across the four VI/CI groups, we report uncorrected *p* values in Tables 2 and 3. Group differences identified in these descriptive analyses should be interpreted with caution, but the *p* values are provided to call attention to potentially meaningful group differences. Third, the associations reported here are based on observational data from which we cannot infer causation. We have attempted to adjust for multiple potential confounders, but it is possible that relationships described herein are explained by unmeasured or unknown confounders.

Future research is needed to understand where opportunities exist to improve care experience and outcomes for the vulnerable subsets of AMI patients described here. While our analysis demonstrates that AMI patients with CI and comorbid CI/VI tend to receive less aggressive revascularization interventions and are less likely to be referred to cardiac rehabilitation postdischarge, it is important to emphasize that we cannot infer whether care decisions were appropriate. Patients who are coping with multiple chronic conditions are underrepresented in research studies and may have different care goals than their younger, healthier counterparts. As a result, strict adherence to guidelines may not always achieve patient-centered, high value care in more medically complex CHD patients. The family members of an older, frail patient with CI and sensory impairment and limited life expectancy may make an informed decision not to pursue catheterization in favor of conservative management and palliation of symptoms. Prospective studies that incorporate both qualitative and quantitative data are needed to elucidate protocols that optimize acute care in this population. For example, seniors with comorbid VI and CI may be excellent candidates for innovative models of care, such as hospital at home,^53,54^ to manage acute presentations of CHD.

In conclusion, almost half of adults ≥75 years old who were hospitalized for AMI were found to have comorbid impairments in cognition, vision, or both. Compared to participants with normal vision and cognition, participants with CI or combined CI/VI were at higher risk of complications during hospitalization and were more likely to experience readmissions and death over the next 180 days. Individuals with concurrent VI and CI had especially high prevalence of comorbidities and disability and were more likely to experience worsened functional status 180 days later. Healthcare teams treating AMI should be prepared to identify and accommodate CI and sensory impairment in older patients and recognize that these conditions may help identify a high risk group. Additional research is needed to determine policies that achieve best short- and long-term outcomes in these medically complicated patients.
